# Characterization of white adipocyte heterogeneity using data-driven microscopy

**DOI:** 10.1038/s41366-026-02151-x

**Published:** 2026-07-08

**Authors:** Jieyu Zhang, Christian Simonsson, Ayesha Fawad, Wathik Alsalim, Allan Vaag, Mathis Neuhaus, Karin G. Stenkula

**Affiliations:** 1 Department of Experimental Medical Science, Lund University, Lund, Sweden; 2 Department of Endocrinology, Skåne University Hospital, Malmö, Sweden

**Keywords:** Cell biology, Obesity

## Abstract

**Background:**

Recent advancements in single-cell transcriptomic analyses have greatly expanded our understanding of cellular diversity in adipose tissue, indicating transcriptionally distinct subpopulations of adipocytes. Here we sought to rigorously test such heterogeneity at the protein level.

**Methods:**

A data-driven imaging platform was tailored to assess cellular protein levels based on immunofluorescence staining in primary adipocytes isolated from human adipose tissue biopsies (abdominal and gluteal adipose tissue from *n* = four subjects). Isolated adipocytes were co-labeled for GLUT4 and the previously proposed adipocyte subtype markers PLIN1 and DDR2 to assess their relative expression levels.

**Results:**

The platform enabled a two-step gating, shape-based filtering approach, which allowed accurate identification of mature adipocytes of a large cell-size range. The software was able to accurately detect hundreds of adipocytes spanning a broad size range. When comparing cells isolated from a single adipose tissue depot and subject, we found that a large proportion of cells had relatively high levels of both DDR2 and PLIN1. No notable differences in GLUT4 levels were observed within this group of cells.

**Conclusions:**

The described approach, based on population-wide imaging, could serve as a robust tool to further validate cellular characteristics at a single-cell level. The fact that we detected distinct protein levels of the proposed adipocyte markers may reflect the presence of mature adipocyte subtypes.

## Introduction

Lately, single-cell sequencing has greatly expanded our understanding of cellular diversity in white adipose tissue. Several studies have identified transcriptionally distinct adipocyte subpopulations that may differentially influence adipose tissue metabolism and whole-body glucose- and energy homeostasis [1–4]. Still, whether these subgroups differ in function (hormone response, lipid metabolism) remains to be explored. Furthermore, the heterogeneity in protein expression of adipocyte subtype markers at the single-cell level has been scarcely described. Here, we used a data-driven imaging platform [5] to assess this in individual primary adipocytes of a large cell-size range isolated from human adipose tissue biopsies. Specifically, we assessed PLIN1 and DDR2 to investigate the abundance of Adipo^PLIN^ and Adipo^LEP^ subgroups [4], in combination with GLUT4 as a marker of insulin sensitivity [6]. We showed that mature adipocytes display distinct protein levels of subtype markers when comparing individual cells isolated from the same adipose tissue depot. Together, this approach could serve as a robust tool for further validation of transcriptional-derived findings at a single-cell level.

## Materials and methods

### Subjects

Adipose tissue biopsies were collected from subjects recruited to the Swedish WC-FAT study, an ongoing study enrolling participants (≥18 years of age with obesity (BMI ≥ 30 kg/m²)) at the Endocrinology and Obesity Unit at Skåne University Hospital (Malmö, Sweden). In this study, we used adipose tissue needle biopsies collected from four subjects (three females and one male, BMI 35.5–50 kg/m^2^) under local anesthesia. All participants provided written informed consent prior to any study procedures. The study was approved by the Swedish Ethical Review Authority 2204-05/26-01, adhering to the revised Declaration of Helsinki.

### Antibodies

Primary antibodies against perilipin-1 #4854 (RRID:AB_2927396) Vala Science (San Diego, USA), GLUT4; LM048 #CSB0148 (RRID:AB_3106913) Cell surface Bio (Philadelphia, USA), and DDR2 PA5-27752 (RRID:AB_2545228), Thermo Fisher Scientific (Waltham, USA). Cross-adsorbed fluorescently conjugated secondary antibodies #A11013 (RRID:AB_2534080), #A11036 (RRID:AB_10563566), #A21236 (RRID:AB_2535805) were from Thermo Fisher Scientific.

### Adipocyte isolation and staining

Primary adipocytes were isolated in Krebs Ringer Bicarbonate HEPES (KRBH) buffer, pH 7.4, containing 200 nM adenosine and 1% (w/v) bovine serum albumin (BSA) using an established protocol [7], cells were washed into phosphate-buffered saline (PBS), and fixed in 4% paraformaldehyde for 7 min. Following fixation, cells were washed in PBS and subsequently blocked for 1 h with buffer containing 1% BSA, 1% goat serum, 0.05% saponin. After blocking, cells were sequentially incubated 1 h with primary antibodies (perilipin-1, 1:300; LM048, 1:500; DDR2, 1:200) and 1 h with secondary antibodies (1:300) diluted in washing buffer with careful washing in between. Finally, cells were resuspended in PBS for image analysis.

### Image analysis and data processing

**Fig. 1:**
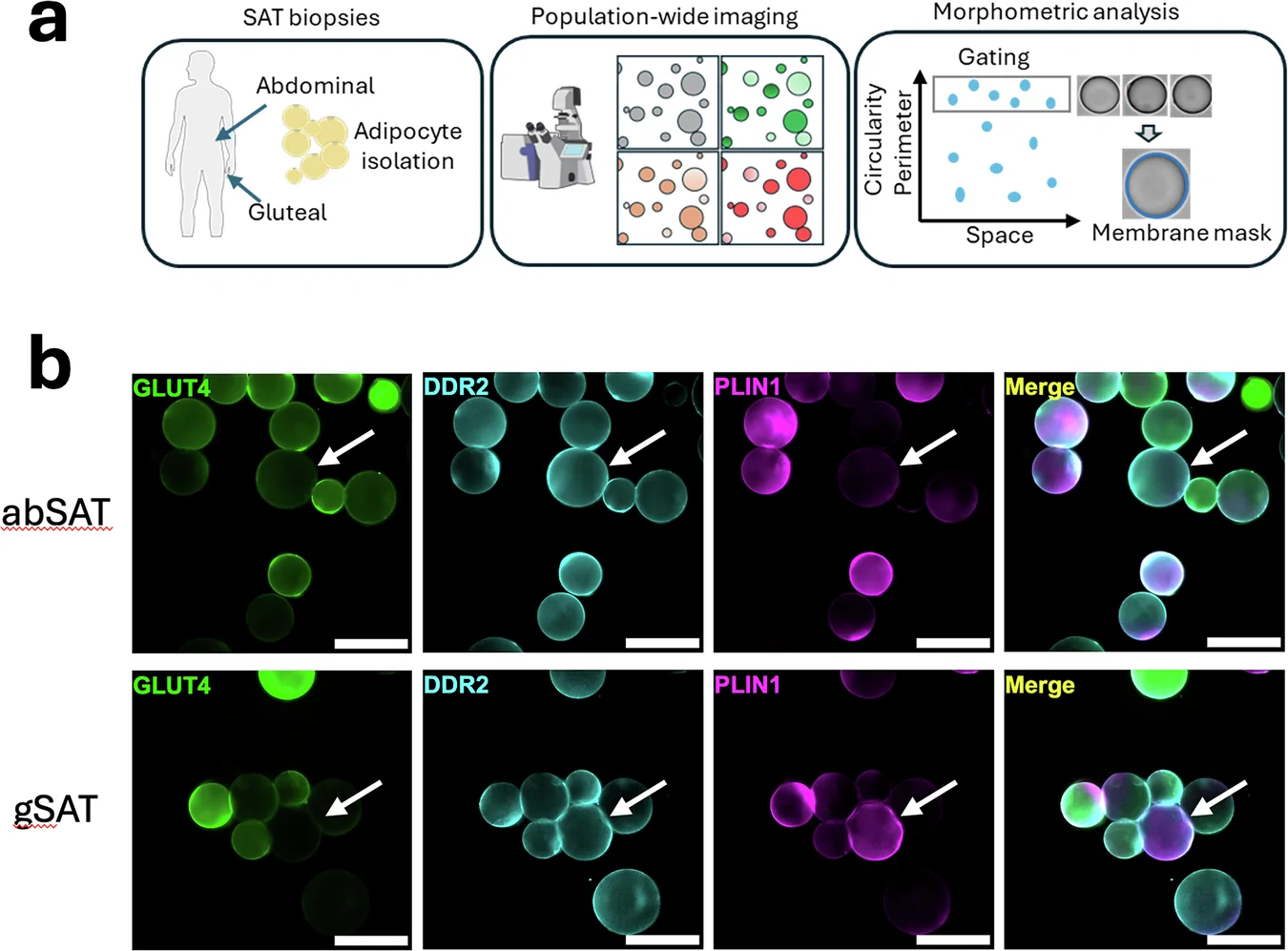
Workflow. **a** Mature adipocytes were isolated from adipose tissue needle biopsies (gluteal and abdominal depots collected from the same subject). Cells were further processed for immunochemistry staining and population-wide imaging using epifluorescence microscopy. Bright-field images were used for segmentation, including gating (circularity and perimeter) to exclude background and distorted signals to allow single-cell intensity measurements. A membrane mask was created to define the region for intensity measurement (see method section for details). **b** Representative images of single adipocytes stained for GLUT4 (green), DDR2 (cyan) and PLIN1 (magenta). Arrows indicate examples of cells showing different signal intensities of GLUT4, DDR2, and PLIN1. Scale bar = 75 µm.

Cells were imaged in petri-dishes (P35G-1.5-20-c, MATTEK) using a Nikon TiE TIRF microscope equipped with a Photometrix Prime 95B sCOMOS camera, a 20× objective with NIS-Elements AR software (version 5.30.07), equipped with 470 nm, 555 nm, and 640 nm lasers. Single-stack images at the widest section of the cells were captured using an automated stage scan setup under NIS-Elements JOBS function. A radius of 2.7 mm was defined as the working area at the center of the actual stage position. The scan direction of the working area was set to “Meander”, touching the frames on the border. A total of 45 acquisition points were generated. The area restriction was set to cover the entire area, ensuring complete coverage of the circular working area. All images were captured using the same laser settings for samples obtained from the same subject. Images were processed using the data-driven microscopy platform (Cytely) [8], where adipocytes were segmented from DIC images using a Laplacian of Gaussian filter and sigma-clipped thresholding. Individual cells were resolved via a Circle Hough Transform and watershed algorithm. Membrane-masks were generated by morphological dilation and erosion of the primary masks to capture the plasma membrane, cytosolic space, and lipid droplet-close region but excluding the lipid droplet (workflow, see Fig. 1a). Two-step gating approach based on circularity and perimeter was applied to filter out background, aggregated, and broken cells. Autofluorescence and non-specific binding were determined to be negligible based on post-hoc validation using a secondary-antibody-only control, assessed by sigma-clipped statistics. Mean intensity was calculated as the integrated intensity within the membrane mask divided by the mask area. Data processing and linear regression analysis were performed using Python version 3.10.0, where the raw intensity data were log10-transformed. The heterogeneity analysis was performed in MATLAB 2023b (Natick, Massachusetts, MathWorks Inc), where raw intensity data was min-max normalized to 0–1 range, separately for each patient, to ensure comparability across samples for subsequent analysis. The regions for sorting into groups were based on the median value of combined data from all subjects.

## Results

**Fig. 2:**
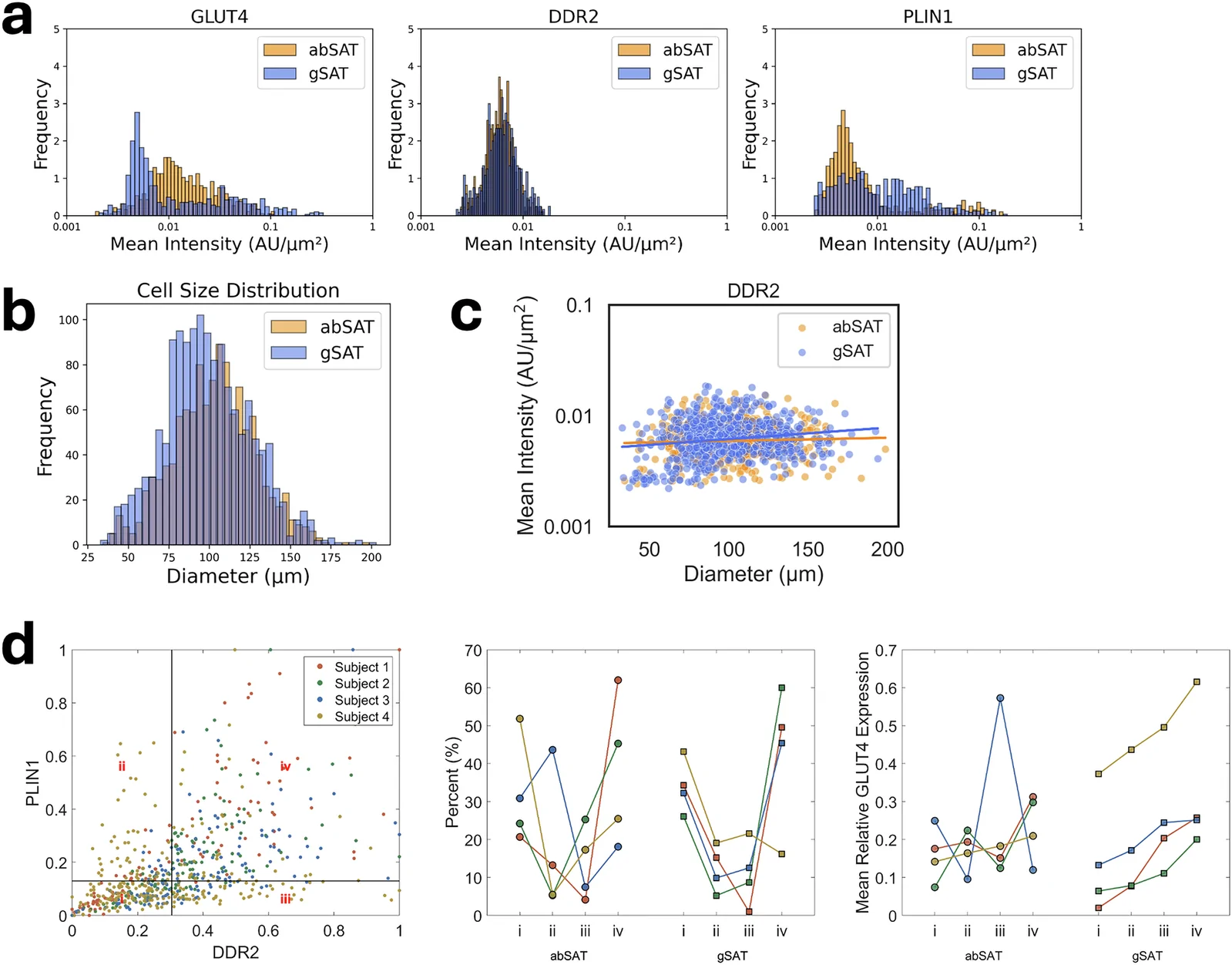
Protein level at a single-cell level. **a** Distribution of cells (frequency, % of total) and mean intensity (mean intensity [AU/µm^2^] = total intensity/ROI) of GLUT4, DDR2 and PLIN1, respectively, retrieved from image analysis. Histograms show combined distribution from four subjects (*n* = 100–200 cells per subject) per adipose tissue depot; abdominal SAT (abSAT, yellow) and gluteal SAT (gSAT, blue). The data presented here are log10-transformed. **b** Cell-size distribution (frequency, number of cells) of detected adipocytes processed for further analysis. Pooled data from four subjects, abdominal adipocytes (abSAT, yellow) and gluteal adipocytes (gSAT, blue). **c** Correlation between DDR2 protein expression level (mean intensity [AU/µm^2^] = total intensity/ROI) and adipocyte size in diameter (µm); abdominal adipocytes (abSAT, yellow); gluteal SAT (gSAT, blue). The mean intensity data presented here are log10-transformed. **d** Correlation between DDR2 and PLIN1 divided into groups based on the median value with low levels defined as below the median, and high level as above the median value resulting in four regions i–iv. Left panel: i Low DDR2/LowPLIN1; ii Low DDR2/High PLIN1; iii high DDR2/low PLIN1; and iv High DDR2/High PLIN1. Middle panel: The dots show the proportion (%) of each subject and depot in each region. Right panel: The dots show the mean relative GLUT4 level for each region within each subject for both depots.

After configuring the image segmentation to detect intact adipocytes, a process including two-step gating to exclude background and artifacts, ~100–200 cells/sample were identified and processed for further analysis. First, we aimed to validate protein expression using selected markers of adipocyte subtypes previously described [1–4]. However, due to poor antibody specificity and the lack of confirmation of mature adipocyte-specific staining, we decided to continue software optimization and data collection using antibodies against DDR2 and PLIN1 (used here as markers of the Adipo^LEP^ and Adipo^PLIN^ subtypes [4], and GLUT4 as a marker of insulin sensitivity. Visual inspection of collected images revealed a clear cell-to-cell variation in labeling intensities, where for example, cells displayed low PLIN1 but relatively strong DDR2 signal (indicated with arrows in Fig. 1b) (images from each depot and subject are shown in Supplementary Fig. S1). Analysis of the entire cell population showed a higher proportion of cells with low GLUT4 expression in gluteal versus abdominal adipocytes, whereas the opposite trend was observed for PLIN1 (Fig. 2a). Similar staining distribution was observed for DDR2 when comparing gluteal and abdominal adipocytes (Fig. 2a) (distribution for each depot and subject are shown in Supplementary Fig. S2). Depot comparison showed intra-subject variations in protein expression when comparing gluteal versus abdominal adipocytes, especially for PLIN1 and GLUT4 (Supplementary Fig. S2). Thoroughly gating of identified objects was performed to assure accurate adipocyte detection. Nevertheless, we were still able to retrieve data from adipocytes spanning a wide range of cell sizes; 45–175 µm diameter in both depots (Fig. 2b). Notably, no correlation between protein level and cell size was identified (example of DDR2 signal versus cell size is shown in Fig. 2c). While we did not perform the data-driven microscopy approach on all markers, we confirmed that the method is suitable for use in primary adipocytes (Supplementary Fig. S3).

Next, to examine possible staining heterogeneity, we compared the relative protein levels of DDR2 and PLIN1. Cells were divided into groups based on the median relative expression value across all subjects, with low expression defined as below the median value, and high as above the median value, resulting in four (i–iv) groups when comparing two protein markers, as illustrated in the left panel of Fig. 2d (i = DDR2 below median/PLIN1 below median, ii = DDR2 below median/PLIN1 above median; iii = DDR2 above median/PLIN1 below median; iv = DDR2 above median/PLIN1 above median). This approach indicated that a moderately large proportion of cells had high levels of both relative DDR2 and PLIN1 (group iv), a similar distribution between groups was shown for all subjects and depots (Fig. 2d, middle panel). Notably, the distribution of GLUT4 level did not follow DDR2 and PLIN1 levels (Fig. 2d, right panel).

## Discussion

In line with recent single-cell transcriptomic studies proposing adipocyte subpopulations based on distinct gene expression [1, 2, 4], we detected distinct protein levels at the single-cell level among cells isolated from the same adipose tissue depot. A simplified classification of the analyzed targets revealed subgroups of cells, where most cells that had high levels of DDR2 also had high levels of PLIN1. However, this subgroup of cells did not have higher GLUT4 levels compared to the other cell groups, which could indicate that there are no differences in insulin sensitivity between these different groups (i–iv). Notably, a large proportion of cells were also classified as having relatively, low overall protein signals, which could be due to data thresholding. Nevertheless, our findings revealed substantial cell-to-cell variation in protein levels of markers that could be related to adipocyte subtypes, possibly originating from distinct stem cell populations, or environmental adaptations during adipocyte maturation. Earlier functional studies describing adipocyte heterogeneity related to insulin response [9, 10], could indeed mirror such subtypes. Surprisingly, we observed considerable variations in PLIN1 levels. PLIN1 deficient adipocytes have been described as degenerative [11], associated with increased inflammation in mice [12], and lipodystrophy in humans [13]. The varying levels observed here may reflect cellular proximity to immune-triggering activities in situ. Overall, our preliminary observations warrant further studies to elucidate the presence and function of mature adipocyte subtypes.

Numerous open-source tools have emerged to identify adipocytes from microscopy-retrieved images, which are useful for determining cellular characteristics [14–16]. While all require optimization to suit the sample type, the benefit of the platform used in the current study is the ability to perform data-driven microscopy, integrating image acquisition and analysis, as well as optional sequential high-resolution imaging of cells with a specific phenotype based on morphometric analysis [17]. Additionally, the platform has proven suitable for detecting changes in GLUT4 translocation in response to insulin at the single cell level (Supplementary Fig. S4). This could facilitate future in-depth characterization of sub-cellular features to resolve cellular diversity. A strength of our approach was the recovery of a broad range of cell-sizes (between 45 and 175 µm in diameter), where usually a narrower range is obtained [16]. Together, our approach provides a straightforward method to systematically and unbiasedly assess single adipocytes within the same sample.

Current advancements in single-nuclei RNA seq analyses require complementary studies to validate cell function and cell identity underlying the gene expression profiles (especially since mature adipocytes account for only 50% of the cell population). The strategy presented herein could support such studies, for example, by enabling the assessment of cellular insulin responsiveness at the single cell level.

## Supplementary information

Supplemental Figure 1

Supplemental Figure 2

Supplemental Figure 3

Supplemental Figure 4

Supplemental Figure legend

## Data Availability

The data generated in Cytely are accessible through a read-only link: https://ref.cytely.io/KX541SQV. The datasets analyzed during the current study are available from the corresponding author on reasonable request.
