# Supplementary material for: Characterization of white adipocyte heterogeneity using data-driven microscopy
Source: Int J Obes (Lond). 2026 Jul 8;50(10):2244–7. doi: 10.1038/s41366-026-02151-x (PMC13722592; doi:10.1038/s41366-026-02151-x)
Supplement: Supplementary file 1 [file 41366_2026_2151_MOESM1_ESM.pdf]

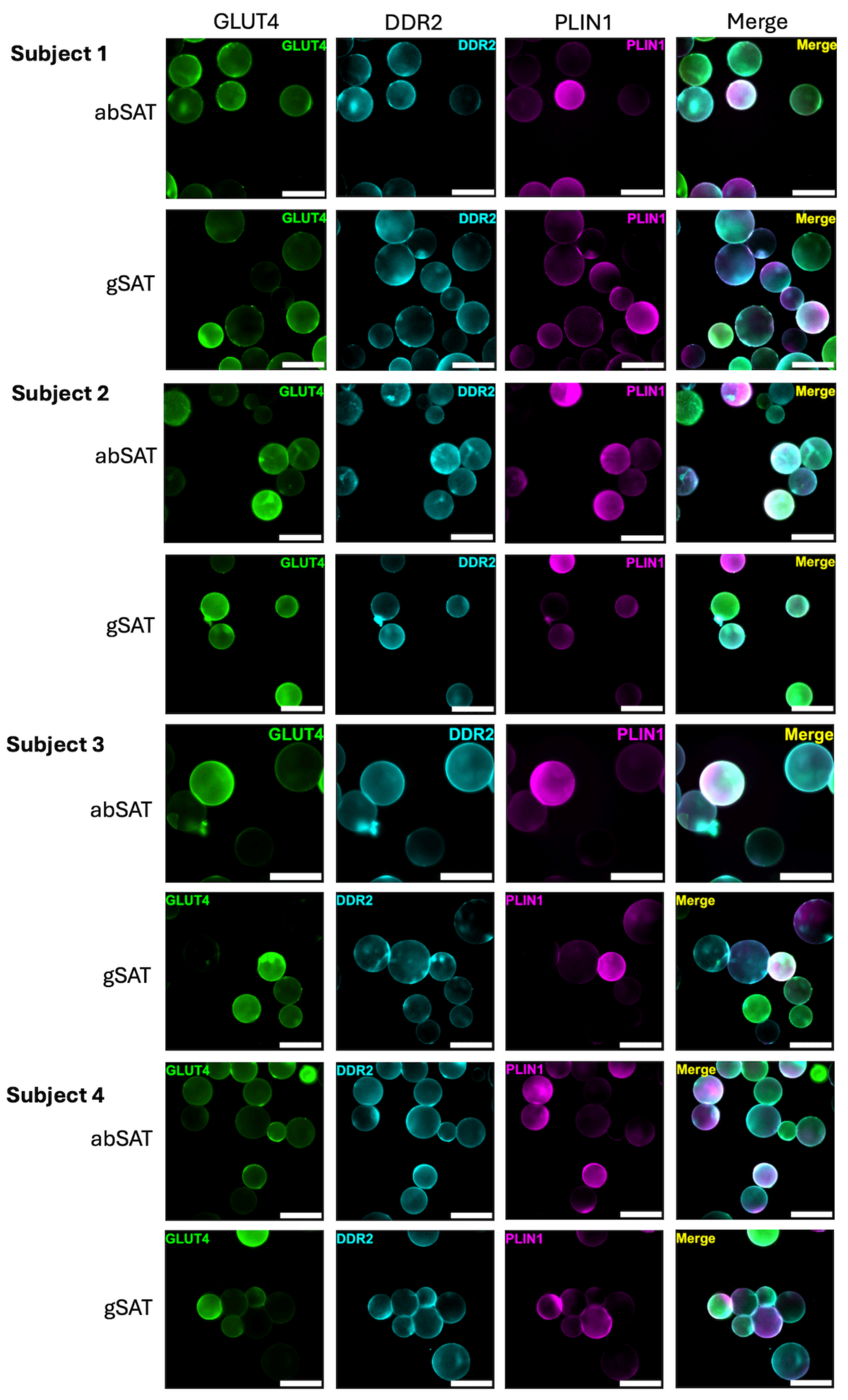
GLUT4
DDR2
PLIN1
Merge
Subject 1
abSAT
gSAT
Subject 2
abSAT
gSAT
Subject 3
abSAT
gSAT
Subject 4
abSAT
gSAT
