# Supplementary material for: Characterization of white adipocyte heterogeneity using data-driven microscopy
Source: Int J Obes (Lond). 2026 Jul 8;50(10):2244–7. doi: 10.1038/s41366-026-02151-x (PMC13722592; doi:10.1038/s41366-026-02151-x)
Supplement: Supplementary file 2 [file 41366_2026_2151_MOESM2_ESM.pdf]

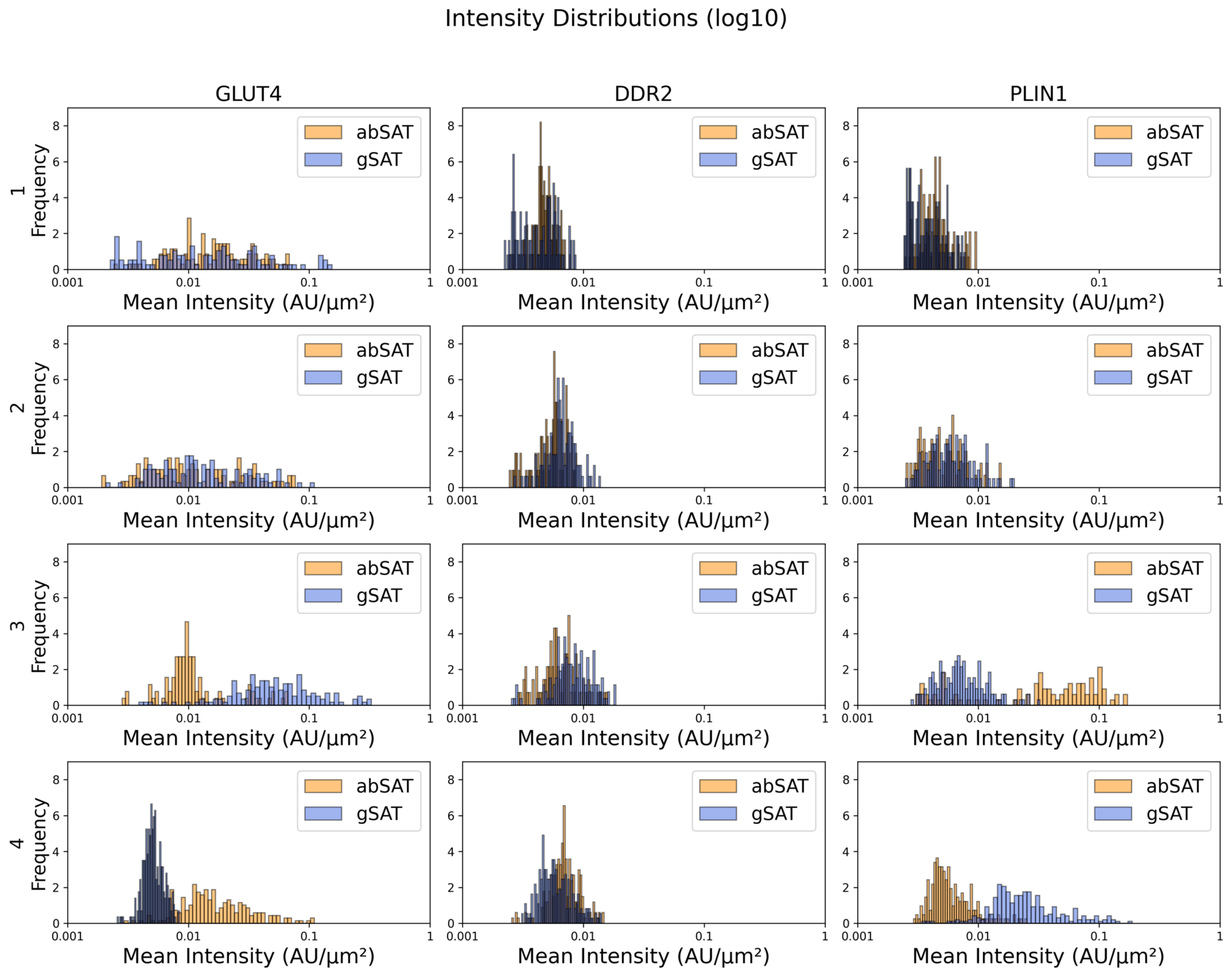

Intensity Distributions (log10)
GLUT4
DDR2
PLIN1
1
2
3
4
Frequency
abSAT
gSAT
Mean Intensity (AU/μm²)
0.001
0.01
0.1
1
0
2
4
6
8
