# Supplementary material for: Characterization of white adipocyte heterogeneity using data-driven microscopy
Source: Int J Obes (Lond). 2026 Jul 8;50(10):2244–7. doi: 10.1038/s41366-026-02151-x (PMC13722592; doi:10.1038/s41366-026-02151-x)
Supplement: Supplementary file 3 [file 41366_2026_2151_MOESM3_ESM.pdf]

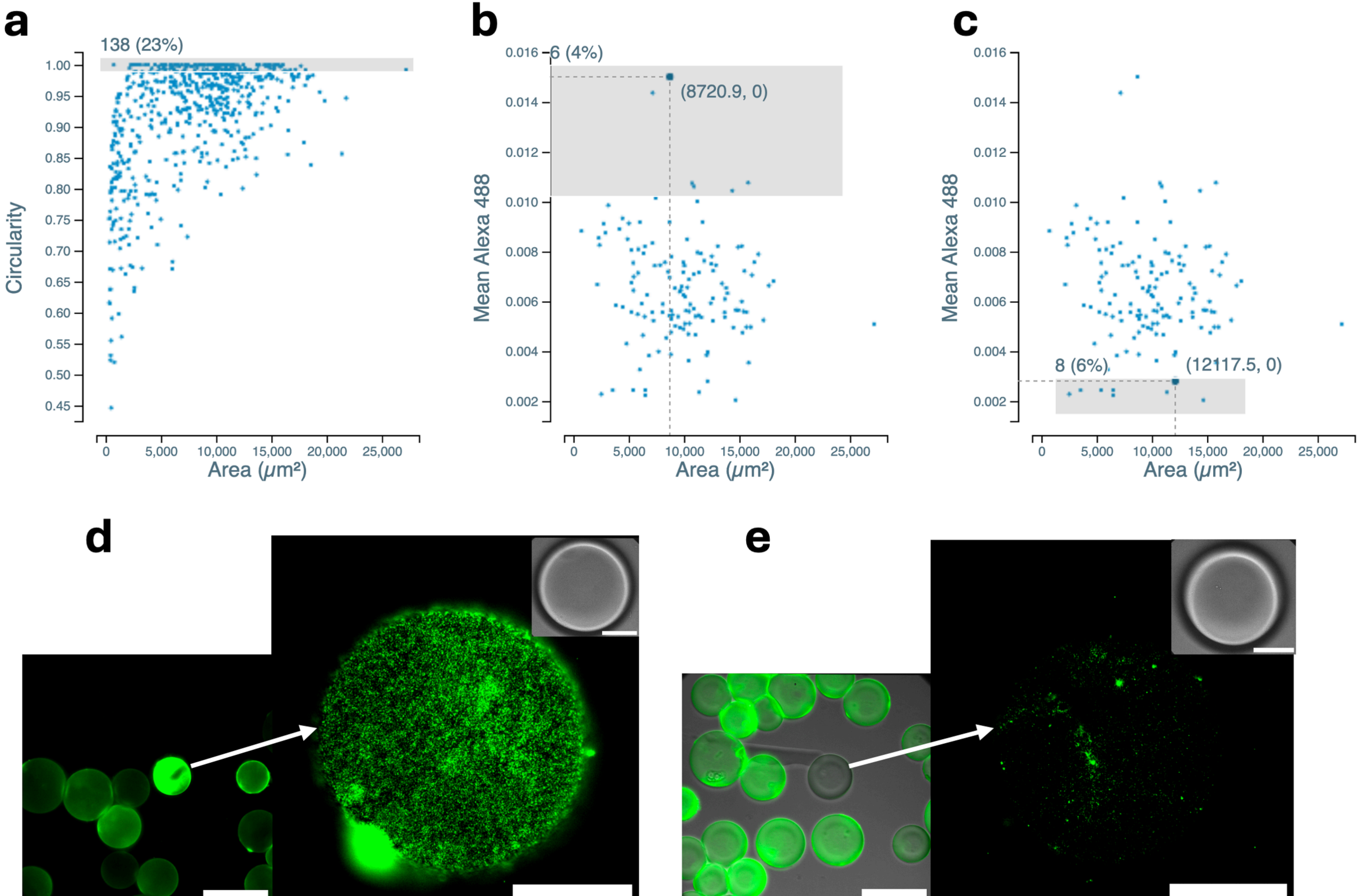

a
138 (23%)
Circularity
Area (μm²)
b
6 (4%)
(8720.9, 0)
Mean Alexa 488
Area (μm²)
c
8 (6%)
(12117.5, 0)
Mean Alexa 488
Area (μm²)
d
e
