# Supplementary material for: Characterization of white adipocyte heterogeneity using data-driven microscopy
Source: Int J Obes (Lond). 2026 Jul 8;50(10):2244–7. doi: 10.1038/s41366-026-02151-x (PMC13722592; doi:10.1038/s41366-026-02151-x)
Supplement: Supplementary file 4 [file 41366_2026_2151_MOESM5_ESM.docx]

Supplementary Figure S1

Representative images of single adipocytes stained for GLUT4 (green), DDR2 (cyan), and PLIN1 (magenta) are shown for abdominal and gluteal adipocytes (abSAT and gSAT) for each subject (1-4).

Supplementary Figure S2

Distribution of cells (frequency, % of total) and mean intensity levels (AU/µm^2^) of GLUT4, DDR2 and PLIN1, respectively, are shown for abdominal and gluteal adipocytes (abSAT and gSAT) for each subject (1-4).

Supplementary Figure S3

Results of data driven microscopy. (a) Scatter plot of object area versus circularity used for morphology-based quality control. Irregular or damaged cells were excluded by applying a circularity threshold (grey bar), retaining 23% of the detected objects for subsequent analysis. (b-c) Scatters plots of mean GLUT4 intensity (referred as Mean Alexa 488 in the plot) versus object area used to identify cells with differential GLUT4 expression. Cells with high (b) or low (c) fluorescence intensity were selected within the grey bar. From here, Cytely was able to save and send the coordinates of these cells highlighted in the grey bar back to the microscope for high-resolution acquisition. (d-e) Representative example of a cell with high GLUT4 expression (d), corresponding to the point marks with dash lines in (b); and a cell with low GLUT4 expression (e), representing the point marks with dash lines in (c). A low-magnification overview image (left, 20x, scale bar = 200µm) is followed by a high-resolution image (right, 100x, scale bar = 50 µm). Contrast correction was performed on the image magnified 100x to improve visual clarity.

Supplementary Figure S4

Representative images of primary adipocytes either non- or insulin stimulated (10 nM, 5 min) prior fixation and labelling with exofacial GLUT4 antibody, scale bar=200 µm. Graph displays exofacial GLUT4 signal (AU/ROI) from individual cells, n=three independent biological replicates, data of mean GLUT4 signal was recently published in Kopietz F et al. (18).

18.	Kopietz F, Neuhaus M, Borreguero-Munoz A, Kryvokhyzha D, Stenkula KG. Focal Adhesion Kinase Orchestrates GLUT4 Translocation and Glucose Uptake via Cytoskeletal Turnover in Primary Adipocytes. FASEB J. 2025;39(10):e70660.
